# Supplementary material for: Witnessing light-driven entanglement using time-resolved resonant inelastic X-ray scattering
Source: Nat Commun. 2023 Jun 14;14:3512. doi: 10.1038/s41467-023-38540-3 (PMC10267212; doi:10.1038/s41467-023-38540-3)
Supplement: Supplementary file 1 — Supplementary Information [file 41467_2023_38540_MOESM1_ESM.pdf]

Supplementary Information: Witnessing Light-Driven Entanglement using Time-Resolved Resonant Inelastic X-Ray Scattering

Jordyn Hales,[1] Utkarsh Bajpai,[1] Tongtong Liu,[2] Denitsa R. Baykusheva,[3] Mingda Li,[4] Matteo Mitrano,[3] and Yao Wang[1]

[1]*Department of Physics and Astronomy, Clemson University, Clemson, SC 29634, USA*

[2]*Department of Physics, Massachusetts Institute of Technology, Cambridge, Massachusetts 02139, USA*

[3]*Department of Physics, Harvard University, Cambridge, Massachusetts 02138, USA*

[4]*Department of Nuclear Science and Engineering, Massachusetts Institute of Technology, Cambridge, Massachusetts 02139, USA*

(Dated: April 27, 2023)

SUPPLEMENTARY NOTE 1: DERIVATION OF THE SELF-CONSISTENT EQUATION

The nonequilibrium dynamic spin structure factor $S(q,\omega,t)$ defined in Eq. (2) of the main text reads as

$$S(q,\omega,t) = \frac{1}{4\pi^2\sigma_{\rm pr}^2 N} \iint\limits_{-\infty}^{+\infty} d\tau d\bar{\tau}\, e^{-(\bar{\tau}-t)^2/\sigma_{\rm pr}^2} e^{-\tau^2/4\sigma_{\rm pr}^2} e^{i\omega\tau} \left\langle \hat{\rho}^{\rm s}_{-q}\left(\bar{\tau}+\frac{\tau}{2}\right) \hat{\rho}^{\rm s}_{q}\left(\bar{\tau}-\frac{\tau}{2}\right)\right\rangle , \tag{1}$$

where we use the Wigner transformation of the time variables $\bar{\tau} = (t_1+t_2)/2$ and $\tau = t_1 - t_2$. In this paper, we employ a pure initial state $|\psi(-\infty)\rangle$ at zero temperature for all simulations, with the aim to reduce the computational complexity and focus on the nonequilibrium aspect of the problem. However, the expressions for the QFI and trRIXS spectra, as well as the relation between them [i.e. Eq. (6) of the main text] are not restricted to a pure state, but can be generalized to any mixed initial state with time-independent distribution weights. Guaranteed by the linearity of the Supplementary Eq. (1), the average notation can be generalized to represent a thermal ensemble

$$\left\langle \hat{O}_1(t_1)\hat{O}_2(t_2)\right\rangle = {\rm Tr}\left[\frac{e^{-\beta\mathcal{H}}}{\mathcal{Z}} \hat{U}(-\infty,t_1)\hat{O}_1\hat{U}(t_1,t_2)\hat{O}_2\hat{U}(t_2,-\infty)\right] . \tag{2}$$

The $\hat{U}(t_1,t_2)$ is the unitary time evolution operator, $\mathcal{Z}$ is the partition function of the equilibrium state (at $t=-\infty$), and β is the inverse temperature. To simplify the derivation, we define the momentum-space spin excitation operator

$$\hat{\rho}^{\rm s}_q = \sum_i \hat{S}^z_i e^{iq\cdot r_i} . \tag{3}$$

Note that the $\hat{\rho}_q$ in Supplementary Eq. (1) is written in the interaction picture as defined in the main text Sec. II and evolves according to the driven EHM Hamiltonian after the Peierls substitution. Therefore, Supplementary Eq. (1) does not obey time-translational invariance and the $\bar{\tau}$ cannot be separated. Integrating in ω the left- and right-hand sides of Supplementary Eq. (1) and Taylor expanding $\hat{\rho}^s_q$ in τ lead to the identity

$$\int_{-\infty}^{+\infty} d\omega\, S(q,\omega,t) = \frac{1}{2\pi N\sigma_{\rm pr}^2} \int_{-\infty}^{+\infty} e^{-\frac{(t-\tau)^2}{\sigma_{\rm pr}^2}} \langle \hat{\rho}^{\rm s}_{-q}(\tau)\hat{\rho}^{\rm s}_{q}(\tau)\rangle\, d\tau. \tag{4}$$

This equality states that the energy integral of the nonequilibrium dynamic spin structure factor at a given momentum q is a convolution of the two-time spin correlation functions with the time-dependent profile of a probe with finite pulse duration. Note that the QFI $f_{\mathrm{Q}}(q,t)$ is defined as $4\langle\hat{\rho}^{\mathrm{s}}_{-q}(t)\hat{\rho}^{\mathrm{s}}_{q}(t)\rangle/N$, when the SU(2) symmetry is preserved. (The disconnected part of Eq. (1) in the main text is non-zero in the presence of long-range magnetic order; however, it can be evaluated separately from the elastic scattering peak intensity and subtracted off from the correlation function). The expression for $f_{\mathrm{Q}}(q,t)$ is an equal-time measurement for an instantaneous wavefunction at time t [1–3]. Therefore, without time-translational invariance in nonequilibrium systems, one cannot obtain the correct time evolution of the QFI by just integrating the dynamic spin structure factor.

However, the information about the time sequence of the QFI $\{f_Q(q,t)| -\infty < t < \infty\}$ is encoded in the entire time evolution of $S(q,\omega,t)$, although without snapshot-to-snapshot correspondence. Therefore, one can apply self-consistent iterations to deconvolve f_Q from the integral equation. By introducing a change of variable $x=\tau-t$ we can Taylor expand $f_Q(\tau)=f_Q(t+x)$ around t in Supplementary Eq. (4) to get

$$\int_{-\infty}^{+\infty} d\omega S(q,\omega,t) = \frac{1}{8\pi\sigma_{\mathrm{pr}}^2}\sum_{m=0}^{\infty}\frac{1}{m!}\frac{\partial^m f_{\mathrm{Q}}}{\partial t^m}\int_{-\infty}^{+\infty} e^{-x^2/\sigma_{\mathrm{pr}}^2}x^m dx. \tag{5}$$

Noting that the integral on the right-hand side is nonvanishing only for even values of m, we can rewrite this equation in the form of Eq. (5) of the main text

$$f_{\mathrm{Q}}(q,t) = 8\sigma_{\mathrm{pr}}\sqrt{\pi}\int_{-\infty}^{+\infty} d\omega\, S(q,\omega,t) + \sum_{m=1}^{\infty}\frac{\mathcal{C}_m}{2m!}\frac{\partial^{2m} f_{\mathrm{Q}}}{\partial t^{2m}}, \tag{6}$$

where $\mathcal{C}_m = -(1/\sigma_{\mathrm{pr}}\sqrt{\pi})\int_{-\infty}^{\infty} e^{-x^2/\sigma_{\mathrm{pr}}^2}x^{2m}dx = -(\sigma_{\mathrm{pr}}^{2m}/\sqrt{\pi})\Gamma(m+1/2)$. Since simulating correlated electrons using the Krylov-subspace requires very small time steps, $S(q,\omega,t)$ is evaluated over a fine time grid and enables the reliable calculation of high-order derivatives.

Supplementary Eq. (6) can be numerically solved by a self-consistent iteration scheme where we truncate the derivative series to some finite $m=M$ and start with the snapshot QFI as

$$f_{\mathrm{Q}}^{(0)}(q,t) = \bar{f}_{\mathrm{Q}}(q,t) = 8\sigma_{\mathrm{pr}}\sqrt{\pi}\int d\omega\, S(q,\omega,t)\,. \tag{7}$$

Then the k-step iteration depends on the lower orders as

$$f_{\mathrm{Q}}^{(k)}(q,t) = 8\sigma_{\mathrm{pr}}\sqrt{\pi}\int_{-\infty}^{+\infty} d\omega\, S(q,\omega,t) + \sum_{m=1}^{M}\frac{\mathcal{C}_m}{(2m)!}\frac{\partial^{2m} f_{\mathrm{Q}}^{(k-1)}}{\partial t^{2m}}, \tag{8}$$

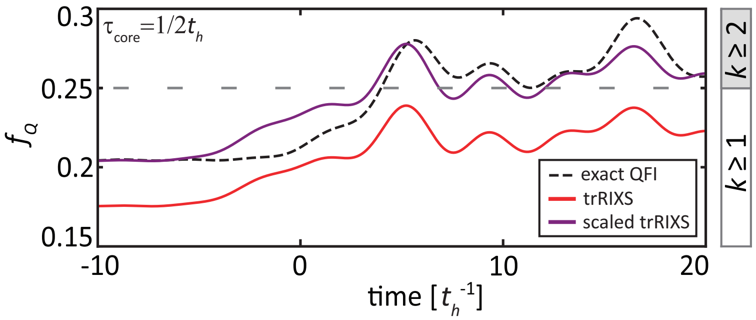

Supplementary Figure 1. QFI dynamics evaluated using the simulated trRIXS with large core-hole lifetime ($\tau_{\rm core} = 1/2t_h$, red curve) and the corrected trRIXS spectra scaled by an overall factor of 1.163 (purple curve) determined by the ratio of equilibrium $S(q,\omega)$ and RIXS, compared with the exact QFI evolution obtained by instantaneous wavefunctions (black dashed curve) .

The convergence is reached by satisfying the criterion $|f_{\rm Q}^{(k)} - f_{\rm Q}^{(k-1)}| < \delta$, where δ is a small number. Once convergence is reached, time-dependent QFI is reasonably well approximated by $f_{\rm Q}(q,t) \approx f_{\rm Q}^{(k)}(q,t)$. In practice, we employ the Fourier method to solve this iterative equation, which does not require an artificial termination.

SUPPLEMENTARY NOTE 2:CORRECTION OF TRRIXS WITH LONG CORE-HOLE LIFETIME

Due to the finite core-hole lifetime, RIXS does not reflect the exact $S(q,\omega)$. As shown in the Fig. 5 of the main text, trRIXS captures the oscillation of QFI for the wavefunction dynamics, but there is a finite offset between the values extracted from trRIXS and those evaluated exactly using the wavefunctions. It is important to note that this offset does not increase in time compared with that at equilibrium. This phenomenon indicates that finite lifetime effect, which causes the deviation between RIXS and $S(q,\omega)$, is insensitive to whether the initial state is equilibrium.

Therefore, we introduce a correction of the trRIXS intensity by a constant factor determined by comparing the equilibrium RIXS spectrum and the $S(q,\omega)$ (which can be measured with inelastic neutron scattering). As shown in Supplementary Fig. 1 for $\tau_{\rm core} = 1/2t_h$, the QFI extracted from equilibrium RIXS spectrum aligns with that from the $S(q,\omega)$ after scaling by an overall factor 1.163. After this rescaling, the QFI extracted from trRIXS is sufficiently accurate throughout the entire dynamics. The efficiency of this correction in

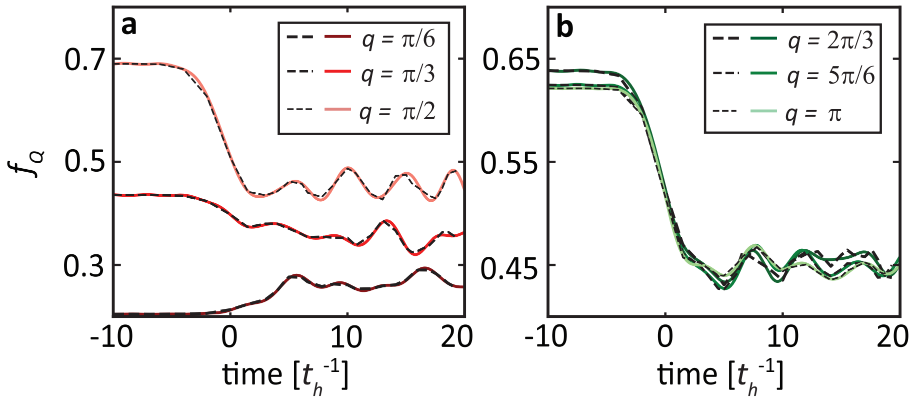

Supplementary Figure 2. QFI density for various momenta. The colored curves represent f_q evaluated from the trRIXS spectra using the self-consistent approach [i.e., main text Eq. (5)]. The corresponding dashed black lines represent the exact QFI density calculated using the instantaneous wavefunction in the main text Eq. (1).

turn demonstrates that trRIXS probes the spin excitations out of equilibrium with limited errors. This statement holds not only for the excitation energy, but also for the spectral intensity after accounting for this constant correction factor.

SUPPLEMENTARY NOTE 3: MOMENTUM DEPENDENCE AND THE EVOLUTION OF THE LOCAL MOMENT

To further test the self-consistent approach, we apply Eq. (5) of the main text to all momenta. As reflected by the comparison between dashed and solid lines in Supplementary Fig. 2, the QFI evaluated from the trRIXS spectra and self-consistent iteration agrees well with that calculated using instantaneous wavefunctions, throughout the entire time evolution. This comparison verifies the general applicability of our approach discussed in Sec. II.

The light-induced doublon-hole fluctuations are reflected by the time-dependent evolution of $\langle\psi(t)|m_z^2|\psi(t)\rangle$, defined as

$$m_z^2 = \sum_i (n_{i\uparrow} - n_{i\downarrow})^2 \,. \tag{9}$$

As shown in Supplementary Fig. 3, the equilibrium local moment at quarter filling is $\langle n\rangle \sim 0.5$. The computed value at negative time delays slightly differs from 0.5 due to the presence of fluctuating double occupancies (0.0076 per site) in the quarter-filled system. At the pump

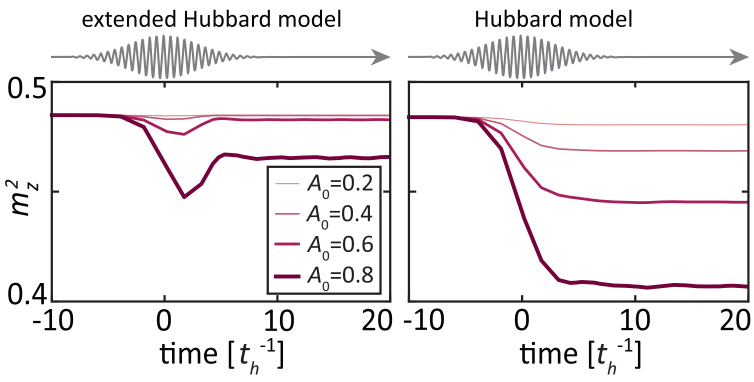

Supplementary Figure 3. Time dependence of the local magnetic moment. Evolution of $\langle m_z^2 \rangle$ in a light-driven EHM (left) and Hubbard model (right) for various pump conditions, as in main text Figs. 6**a**,**b**.

arrival, the light-driven motion of charge carriers generates additional doublons and holes and reduces the magnetic moments. Different from the Hubbard model ($V = 0$), the magnetic moments are largely preserved in the EHM due to the presence of a nonlocal attractive interaction V, as shown in Supplementary Fig. 3. Since this interaction favors adjacent singly-occupied states over doublons and holes, the local moment $\langle m_z^2 \rangle$ quickly recovers after the pump for the EHM, in contrast to the persistent decrease observed in the Hubbard model.

This post-pump recovery of magnetic moments causes the transfer of spin fluctuations among different wavevectors. As shown in the black dashed curves of Supplementary Fig. 2, the compression of spin fluctuations at large momenta, inherent from the equilibrium Luttinger instability, is partially compensated by the enhancement at small ones ($q = \pi/6$ in this system) in the EHM. Such an enhancement is minor for the Hubbard model, as shown in the main text Fig. 6**c**. Therefore, the presence of the nonlocal interactions and the proximity to a phase boundary are crucial for the light-induced entanglement.

SUPPLEMENTARY NOTE 4: NONEQUILIBRIUM ENTANGLEMENT ENTROPY

In this section, we compare the nonequilibrium QFI dynamics with the entanglement entropy, which has been widely employed as an entanglement measure in quantum information. We consider the 1D extended-Hubbard model with the same parameters as the main

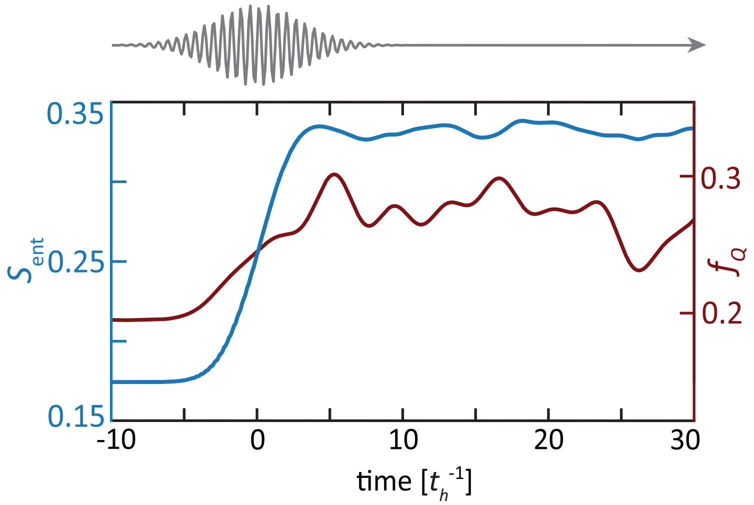

Supplementary Figure 4. Comparison between transient entanglement entropy and QFI density for a light-driven extended-Hubbard model. Model parameters ($U = 8t_h$ and $V = -t_h$) and pump conditions ($A_0 = 1$ and $\Omega = 10t_h$) are the same as those in Fig. 4 of the main text.

text. The Rényi entropy for an instantaneous nonequilibrium state is defined as

$$\mathcal{S}_{\text{ent}}(t;\alpha) = \frac{1}{1-\alpha} \ln \text{Tr}_A \Big[\text{Tr}_{A^c} \left|\psi(t)\right\rangle \left\langle\psi(t)\right| \Big]^\alpha . \tag{10}$$

Here we consider the case $\alpha = 1$. The label A refers to the label of the subsystem A, defined as half of the chain, and A^c refers to the remaining subsystem.

In order to compare the time-dependent entanglement entropy with the QFI dynamics, we adopt the same pump condition as Fig. 4 of the main text, i.e. $A_0 = 1$ and $\Omega = 10t_h$. As shown in Supplementary Fig. 4, the nonequilibrium entanglement entropy increases at the same time of the light-driven QFI, further supporting the notion that the nonequilibrium state exhibits light-enhanced entanglement. It is, however, worth noting that, unlike the QFI, the entanglement entropy is not experimentally accessible in spectroscopic measurements of quantum materials.

SUPPLEMENTARY NOTE 5: UPPER BOUND ON THE QFI BASED ON THE SINGLE-PARTICLE FERMIONIC MODES

In this appendix, we follow Ref. 4 and derive the QFI bounds based on single-particle fermionic modes (SPFM). For the 1D extended Hubbard model of N sites (with periodic boundary conditions) considered in the main text, the operator ρ^{s}_q in Supplementary Eq. (3)

obtained as a linear superposition of local operators S^z_i is non-hermitian at all momenta ($\rho^{s\dagger}_q = \rho^s_{-q}$ except $q = 0, \pi$). The corresponding Hermitian operator can be chosen as $O_q = (\rho^s_q + \rho^s_{-q})/2N$, such that

$$O_q = \frac{1}{N} \sum_{j,\sigma} s_\sigma \cos(jq)\, c^\dagger_{j\sigma} c_{j\sigma}. \tag{11}$$

where $s_\uparrow = +, s_\downarrow = -$. The selection of local density operators in O_q avoids ambiguities related to fermion anticommutation and sign. The fluctuation of this O_q operator is related to the (instantaneous) QFI of the spin operator ρ^s_q through

$$4\langle O_q^2 \rangle = \frac{1}{N} \langle\, (\rho^s_q)^2 + (\rho^s_{-q})^2 \rangle + \frac{f_Q(q,t)}{2}. \tag{12}$$

For the simulated translational-symmetric system in Sec. IV, the time-dependent wavefunction $|\psi(t)\rangle$ has conserved momentum quantum number. This implies that $\langle (\rho^s_q)^2 \rangle \neq 0$ only when $q = 0, \pi$. At the same wavevectors $q = 0, \pi$, ρ^s_q is Hermitian, therefore Supplementary Eq. (12) leads to

$$f_Q(q,t) = \frac{8\langle O_q^2 \rangle}{(1 + \delta_{q0} + \delta_{q\pi})}. \tag{13}$$

To estimate the upper bound on $f_Q(q,t)$, we consider the upper bound on $\langle O_q^2 \rangle$ as derived in Ref. 4 for a fermionic many-body k-producible pure quantum state. For the creation (annihilation) operators $c^\dagger_{n\sigma}$ ($c_{n\sigma}$), all SPFMs are spanned by $n = 1, 2, \cdots N$ and spin $\sigma = \uparrow, \downarrow$. If $P_m = [(n^m_1, \sigma^m_1), (n^m_2, \sigma^m_2), \cdots, (n^m_{N_m}, \sigma^m_{N_m})]$ denotes a unique partition that contains N_m SPFMs, then $\mathcal{P} = P_1 \cup P_2 \cup \cdots \cup P_{N_{\text{parts}}}$ (where $N_{\text{parts}} \leq 2N$ is the number of partitions) forms the complete set of SPFMs. With this notation, Ref. 4 shows that if $|\Psi(t)\rangle_{k-\text{prod}}$ has a fixed number of electrons N_e, then the lower bound on $\langle O_q^2 \rangle = {}_{k-\text{prod}}\langle \Psi(t)|O_q^2|\Psi(t)\rangle_{k-\text{prod}}$ can be determined by choosing a partition $\bar{\mathcal{P}} = \bar{P}_1 \cup \bar{P}_2 \cup \cdots \bar{P}_{N_{\text{parts}}}$ which maximizes the right-hand side of the following inequality

$$\langle O_q^2 \rangle \leq \frac{1}{4} \sum_{m=1}^{N_{\text{parts}}} \left[\sum_{\beta \in P^{\text{high}}_m} A_\beta(q) - \sum_{\beta \in P^{\text{low}}_m} A_\beta(q) \right]^2, \tag{14}$$

where $A_\beta(q) \in \{\sigma \cos(qn) | \sigma = \pm 1, n = 1, 2, \cdots, N\}$ such that $A_1(q) \geq A_2(q) \geq \cdots \geq A_{2N}(q)$ and $\bar{P}_m = P^{\text{high}}_m \cup P^{\text{mid}}_m \cup P^{\text{low}}_m$ represents a decomposition of the partition $\bar{P}_m$. The right-hand side in Supplementary Eq. (14) can be numerically evaluated via the algorithm presented in Ref. 4, assuming conserved particle number N_e. Thus, the upper bound of QFI based on

SPFM can be obtained by substituting Supplementary Eq. (14) into Supplementary Eq. (13), which is the fermionic bound mentioned at the end of Sec. V.

We note that Supplementary Eq. (14) does not automatically impose SU(2), translational, and point-group symmetries, which are challenging to implement in sizeable fermionic systems. Therefore, this bound is usually not strict for doped fermionic systems and for 2-producible SPFM states we find it to be ~ 1.5, i.e. much higher than the value obtained for the spin modes in the main text Fig. 6. Crucially, since the QFI bounds represent a sufficient but not necessary condition for the presence of entanglement, the overall scale of the QFI cannot exclude the presence of other highly-entangled states and the entanglement depth should be estimated via the highest available k witnessed by different QFIs. A more accurate and basis-independent definition of entanglement in fermionic systems requires considering indistinguishable particles subject to anticommutation relations, and the superselection rule [5, 6]. Entanglement measures satisfying these conditions, such as the the Slater rank [7, 8], usually require high-order correlation functions beyond the ones accessible with spectra in the form of the main text Eq. (2). Therefore, distilling entanglement in the fermionic basis from spectroscopic measurements remains an open and interesting question.

[1] L. Pezzé and A. Smerzi, *Entanglement, Nonlinear Dynamics, and the Heisenberg Limit*, Phys. Rev. Lett. **102**, 100401 (2009).

[2] P. Hyllus, W. Laskowski, R. Krischek, C. Schwemmer, W. Wieczorek, H. Weinfurter, L. Pezzé, and A. Smerzi, *Fisher Information and Multiparticle Entanglement*, Phys. Rev. A **85**, 022321 (2012).

[3] G. Tóth, *Multipartite Entanglement and High-precision Metrology*, Phys. Rev. A **85**, 022322 (2012).

[4] R. C. de Almeida and P. Hauke, *From Entanglement Certification with Quench Dynamics to Multipartite Entanglement of Interacting Fermions*, Phys. Rev. Research **3**, L032051 (2021).

[5] H. M. Wiseman and J. A. Vaccaro, *Entanglement of Indistinguishable Particles Shared Between Two Parties*, Physical review letters **91**, 097902 (2003).

[6] M.-C. Bañuls, J. I. Cirac, and M. M. Wolf, *Entanglement in Fermionic Systems*, Phys. Rev. A **76**, 022311 (2007).

[7] J. Schliemann, J. I. Cirac, M. Kuś, M. Lewenstein, and D. Loss, *Quantum Correlations in Two-fermion Systems*, Phys. Rev. A **64**, 022303 (2001).

[8] K. Eckert, J. Schliemann, D. Bruß, and M. Lewenstein, *Quantum Correlations in Systems of Indistinguishable Particles*, Ann. Phys. **299**, 88 (2002).
